# Using Support Vector Machines with Multiple Indices of Diffusion for Automated Classification of Mild Cognitive Impairment

**DOI:** 10.1371/journal.pone.0032441

**Published:** 2012-02-23

**Authors:** Laurence O'Dwyer, Franck Lamberton, Arun L. W. Bokde, Michael Ewers, Yetunde O. Faluyi, Colby Tanner, Bernard Mazoyer, Desmond O'Neill, Máiréad Bartley, D. Rónán Collins, Tara Coughlan, David Prvulovic, Harald Hampel

**Affiliations:** 1 Department of Psychiatry, Psychosomatic Medicine and Psychotherapy, Goethe University, Frankfurt, Germany; 2 Centre for Imaging-Neurosciences and Applications to Pathologies, UMR 6232, CNRS, CEA, University of Caen and Paris Descartes, Caen, France; 3 Cognitive Systems Group, Discipline of Psychiatry, School of Medicine and Trinity College Institute of Neuroscience (TCIN), Trinity College Dublin, Dublin, Ireland; 4 Department of Radiology, VA Medical Center, University of California San Francisco, San Francisco, California, United States of America; 5 Liaison Psychiatry Service, Addenbrooke's Hospital, Cambridge, United Kingdom; 6 Cambridgeshire and Peterborough NHS Foundation Trust, Cambridge, United Kingdom; 7 Department of Zoology, Trinity College Dublin, Dublin, Ireland; 8 Centre Hospitalier Universitaire, Caen, France; 9 Institut Universitaire de France, Paris, France; 10 Department of Medical Gerontology, Trinity College Dublin, Dublin, Ireland; University of Maryland, College Park, United States of America

## Abstract

Few studies have looked at the potential of using diffusion tensor imaging (DTI) in conjunction with machine learning algorithms in order to automate the classification of healthy older subjects and subjects with mild cognitive impairment (MCI). Here we apply DTI to 40 healthy older subjects and 33 MCI subjects in order to derive values for multiple indices of diffusion within the white matter voxels of each subject. DTI measures were then used together with support vector machines (SVMs) to classify control and MCI subjects. Greater than 90% sensitivity and specificity was achieved using this method, demonstrating the potential of a joint DTI and SVM pipeline for fast, objective classification of healthy older and MCI subjects. Such tools may be useful for large scale drug trials in Alzheimer's disease where the early identification of subjects with MCI is critical.

## Introduction

Mild cognitive impairment (MCI) is an intermediate state between healthy aging and Alzheimer's disease (AD), characterised as a non-disabling disorder that represents an early state of abnormal cognitive function [1]. Although not all MCI cases represent prodromal AD, an estimated 10–15% of MCI subjects enter the dementia spectrum every year. In contrast, 1–2% of healthy older people convert to AD each year [1]. Therefore, MCI is frequently considered to be a good target for the early diagnosis of AD [1], [2]. Future drugs for AD, such as amyloid-modifying compounds, may fail to affect the clinical course of AD when neurodegenerative processes are well established, but it has been suggested that these drugs may have greater success in the very earliest stages of AD before the onset of symptoms [3]. Therefore, fast and objective tools for the diagnosis of MCI will be of great interest for future research into the understanding of MCI and AD, as well as for drug development in AD. Existing cognitive batteries which are used for the diagnosis of MCI and AD such as the CERAD [4] are both subjective and extremely time consuming.

Here we wish to develop a method of combining diffusion tensor imaging (DTI) together with support vector machines (SVMs) [5] which may be used to supplement existing cognitive batteries during the diagnosis procedure. DTI probes white matter (WM) structure by exploiting the fact that water diffuses faster along the main axis (λ_1_) of fibers compared with diffusion perpendicular to fibers (λ_2,_ λ_3_) [6]. Four primary indices of diffusion can be assessed – fractional anisotropy (FA), mean diffusion (MD), axial diffusion (DA) and radial diffusion (DR) [7].

Although WM damage has been found in AD both in post-mortem studies [8] and *in vivo* studies [9] little attention has been focused on the potential of using DTI tools to classify MCI and AD subjects. However, this is likely to prove a fruitful area of research as WM damage may be a key indicator of early AD pathology [10].

To date, machine learning techniques have been applied to a range of MRI modalities in an effort to automate the diagnosis of MCI and AD. This includes, the use of volumetric analysis of the hippocampus combined with logistic regression [11] as well as the combination of support vector machines (SVMs) with grey matter (GM) data from voxel based morphometry (VBM) [12], [13]. A combination of structural MRI with PET data has been found to increase accuracy when using SVMs [14]. Risk scores for MCI conversion to AD have been created with VBM data using principal component analysis (PCA), structural equation modelling (SEM) and SVM approaches [5], [12], [13], [15], [16]. Cortical thickness studies have been used to classify AD and control scans [17] while cross-sectional pattern analysis studies have been used to classify control and MCI subjects [18]. Machine learning techniques have also proved to be effective for the classification of MCIs which convert to AD at follow-up and those that remain stable [19], [20].

The aim of the current study was to investigate how multiple indices of diffusion can be used in conjunction with SVMs for the classification of control and MCI subjects. We wanted to assess the efficacy of each index of diffusion for classification. We also wanted to assess the locations of the voxels that were most useful for discriminating between groups. We hypothesized that the most useful voxels for classification would be located in areas that are known to be compromised in the early stages of AD. Previous studies have indicated that atrophy in the early stages of MCI and AD are subtle and distributed in a number of regions including the hippocampus, the lateral and inferior temporal structures, the anterior and posterior cingulate, the uncinate fasciculus and the superior longitudinal fasciculus [21]–[23].

## Methods

### Ethics Statement

The study was approved by the St. James' Hospital and Adelaide & Meath Hospital incorporating the National Children's Hospital Research Ethics Committee and was in accordance with the Declaration of Helsinki. All participants provided informed written consent.

### Participants

Scans were obtained from three groups of participants: 40 healthy older people, 19 MCIna, 14 MCIa. The total number of participants was 73. MCI patients were diagnosed using criteria for both amnestic and non-amnestics sub-groups [24]. Neuropsychological assessment consisted of the Mini Mental State Examination (MMSE) [25] and the Consortium to Establish a Registry for Alzheimer's Disease (CERAD) neuropsychological battery [4]. For the diagnosis of MCI, the following must be present:

1. objective impairment on any neuropsychological test from the CERAD battery based on a cut-off of −1.5 SD below published normative data corrected for age and education of the subject;
2. cognitive impairment corroborated by a close family member;
3. essentially normal activities of daily living;
4. must not meet criteria for dementia as defined below.

MCI individuals with objective memory impairment were diagnosed as having MCIa and those with non-memory impairment were diagnosed as having MCIna.

Diagnostic criteria of AD were that of the National Institute of Neurological Disorders and Stroke–Alzheimer Disease and Related Disorders (NINCDS–ADRDA) working group [26]. MCIna and MCIa participants were recruited at the Adelaide and Meath Hospital incorporating the National Children's Hospital (AMNCH), Dublin, Ireland. Healthy control participants were recruited among relatives of MCI subjects and also through advertisements in the local community.

Participants were excluded if they had cortical infarction, excessive subcortical vascular disease, space-occupying lesions, depression, and any other psychiatric or neurological disease. Participants were also excluded on magnetic resonance imaging criteria such as pacemaker implant, recent metallic implants, and claustrophobia. The DTI and structural scans of the cohort used in the current study were previously used in a study of mixed-effects models [27] and in a study of the role of multiple indices of diffusion in MCI and AD [21].

### Imaging Methods

Magnetic resonance imaging (MRI) was conducted with a Philips Achieva 3.0 Tesla MR system (Best, The Netherlands). A parallel SENSitivity Encoding (SENSE) approach was used. The high resolution 3D T1-weighted structural images were achieved with the following pulse sequence: TR = 8.4 ms; TE = 3.9 ms; flip angle = 8°; number of axial slices = 180; slice thickness = 0.9 mm; acquisition voxel size = 0.9×0.9×1.8 mm^3^; rec voxel size = 0.9×0.9×0.9 mm^3^; field of view (FOV) = 230 mm×230 mm×230 mm; acquisition matrix = 256×256; SENSE reduction factor = 2.3; total acquisition time = 5 min 44 sec.

DTI was acquired using an echo planar imaging (EPI) sequence with the following pulse sequence: TR = 12396 ms; TE = 52 ms; acquisition voxel size = 2×2×2 mm^3^; rec voxel size = 1.75×1.75×2 mm isotropic, 60 axial adjacent slices; slice thickness = 2 mm (no gap); FOV = 224 mm×224 mm×120 mm; acquisition matrix = 112×112; SENSE reduction factor = 2, combined with a half-scan acquisition; 1 image without diffusion weighting and 15 diffusion-encoding gradients applied in 15 noncollinear directions; b-value = 800 s/mm^2^; both the b0 and the 15 diffusion weighted images were averaged twice, bandwidth = 2971 Hz/pixel; total acquisition time = 7 min 34 sec.

A T2-weighted fluid attenuation inversion recovery (FLAIR) sequence was also acquired to ensure that vascular pathology was not significant. All images were rated using the Fazeka scale [28]. The mean and SD for all participants was 1.33, SD: 0.71; while specific subgroups were as follows; Controls: 1.18, SD 0.51; MCIa: 1.08, SD 0.28; MCIna: 1.37, SD 0.83.

### DTI Processing

DTI analysis was performed using TBSS [29]. Images were skull stripped with the Brain Extraction Tool (BET) from the FSL library [30]. Raw DTI images were first corrected for motion and eddy current effects. The diffusion tensor was then calculated with the DTIFIT program for whole brain volumes and the resulting FA maps, together with the DA (λ1) and DR ((λ2+λ3)/2) and MD ((λ1+λ2+λ3)/3) maps, were used in subsequent TBSS analysis.

TBSS performs a non-linear registration that aligns each FA image to every other one and calculates the amount of warping needed for the images to be aligned. The most representative image is determined as the one needing the least warping for all other images to align to it. The FSL library also provides a 1 mm isotropic FA target image (FMRIB58_FA) in standard space, which is sometimes used instead of the most representative image from the study cohort. This can be problematic as the target image is based on a young healthy brain. Using the method of “all subject to all subject” registration is more computationally intensively, but highly desirable when dealing with populations other than young healthy controls.

After this registration step, warped versions of each subject's FA image were generated which were then averaged and a white matter “skeleton” was then created suppressing all non-maximum FA values in each voxel's local-perpendicular direction and subsequently comparing all remaining non-zero voxels with their nearest neighbours, thus searching for the centre of fibre bundles. The skeleton was then thresholded at an FA value of 0.2 which limits the effects of poor alignment across subjects and ensures that GM and CSF voxels are excluded from the skeleton. The resulting skeleton contained WM tracts common to all subjects. A “distance map” is then created which is used to project each FA image onto the mean FA skeleton that is common to all subjects [29]. The same non-linear transformations derived for the FA maps were applied to the DA, DR and MD maps.

Following TBSS processing, a global region of interest was created using the white matter skeleton that is common to all subjects. Mean values of FA, DA, DR and MD were extracted from each subject using this global ROI in order to generate boxplots for control, MCIna and MCIa groups for each index of diffusion.

### SVM Classification Analysis

Classification of individual subjects was undertaken using the freely available WEKA software package (http://www.cs.waikato.ac.nz/ml/weka, Version 3.6.4) [31], [32]. Following TBSS analysis, the skeletonised FA, DA, DR and MD data was analysed in Matlab (program written by FL and available on request), which extracted the diffusion values from the WM skeleton and transformed them into a WEKA compatible format. There were 130,394 voxels in the WM skeleton and diffusion values for all indices of diffusion were extracted from each voxel in the WM skeleton. Classification between groups was undertaken using each index of diffusion separately in order to determine the most efficient index for classification.

Analysis was carried out for two types of classifications:

1. Control and MCI classification
2. Control, MCIa and MCIna classification

The first step of the WEKA analysis was to reduce the number of voxels to those that are most relevant for classification. This step eliminates non-discriminative voxels which would reduce classification accuracy. The feature selection algorithm “ReliefF” [33] was used to extract the most important voxels from the full FA, DA, DR and MD datasets that contain diffusion values from every voxel in the entire white matter skeleton of each subject. For each classification group and also for each index of diffusion, seven reduced datasets were created as follows:

1. 100 voxel dataset
2. 250 voxel dataset
3. 500 voxel dataset
4. 750 voxel dataset
5. 1000 voxel dataset
6. 2000 voxel dataset
7. 3000 voxel dataset.

Therefore in total, 14 reduced datasets were created; i.e. 7 reduced datasets for Control and MCI classification, and 7 reduced datasets for Control, MCIa, and, MCIna classification. The choice of the size of these reduced datasets is based on previous work using a similar approach to the one outlined in the current study [20], [34]. To date, ∼500–1000 voxels have been found to give optimal classification results.

The aim of the ReliefF algorithm is to estimate the quality of voxels according to how well the value of a voxel distinguishes between instances that are near to each other. The algorithm works on the assumption that the voxels of nearby individuals with different diagnoses are the most useful for assessing the predictive ability of the voxel. The current method employs feature selection on the entire dataset which has been used in previous studies [20], [34] while other studies have employed nested cross validation [35], [36]. See the discussion for a note on this point.

**Figure 1:**
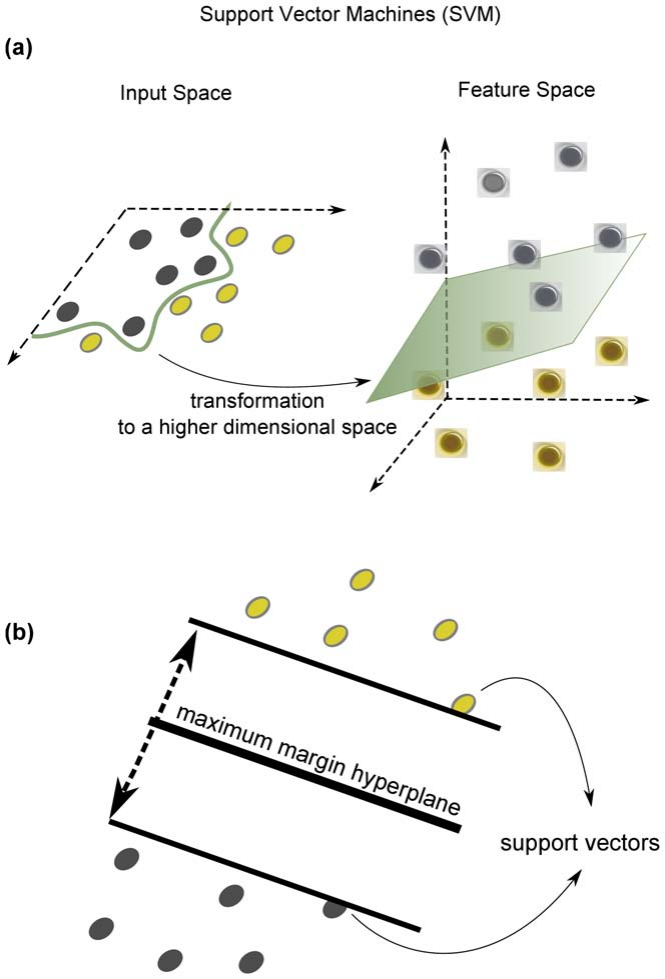
Principle of support vector machines. (a) The algorithm tries to find a boundary that maximises the distance between groups. When the input data is viewed in two-dimensions it cannot be separated by a straight line. However, if the two-dimensional space is transformed into a three dimensional space, then it is possible to separate the data using a hyperplane. (b) The SVM tries to find a boundary that maximizes the distance between groups. The data that are closest to the maximum margin hyperplane are called support vectors. A unique set of support vectors defines the maximum margin hyperplane for the learning problem.

After reducing the data into datasets of differing sizes, classification was then performed using the SVM algorithm “sequential minimal optimization” (SMO) [37] with a radial basis function (RBF) kernel [38]. SVMs are algorithms that learn how to assign labels to objects [5]. They use linear models to implement nonlinear class boundaries by transforming the input into a new higher dimensional space (Fig. 1a). In this way, a straight line in the new space can be curved or non-linear when transformed back to the original lower-dimensional space (Fig. 1a). Following transformation, a linear model called the maximum margin hyperplane is created. To visualise this, imagine a dataset with two-classes that are linearly separable. The maximum margin hyperplane is the one that gives the greatest separation between the classes. The hyperplane describes a straight line in a high-dimensional space, and therefore a separating hyperplane is a line that separates the classes (see Fig. 1b). The instances that are closest to the maximum margin hyperplane are called support vectors. A unique set of support vectors defines the maximum margin hyperplane for the learning problem. Once the support vectors are established, a maximum margin hyperplane can be constructed. The maximum margin hyperplane is relatively stable as it only moves if the training instances that are added or deleted are support vectors. This holds true in high-dimensional space spanned by the nonlinear transformation. Support vectors are usually few in number which gives little flexibility and thus guards against overfitting which can arise when there is too much flexibility in a decision boundary.

The projection of the data from low dimensional space to higher dimensional space is achieved with a kernel function. The optimal kernel function is usually found by trial and error. In the current study a radial basis function (RBF) kernel was used to nonlinearly map samples into a higher dimensional space. RBF kernels use two parameters: C and GAMMA. GAMMA represents the width of the radial basis function, and C represents the error/trade-off parameter that adjusts the importance of the separation error in the creation of the separation surface. C was fixed to 1 and GAMMA was fixed to 0.01.

Once the SVM has been trained, a new test subject can be labelled, based on the distance between the subject and the separating hyperplane. The distance is used by the classifier to determine, via Platt's method [39], the probabilistic score for the subject and the subject is labelled based on the sign of the score. Platt's method uses a sigmoid function to enable receiver operating characteristic (ROC) curves to be generated. The approach applied here is to train an SVM first, and then to train the parameters of an additional sigmoid function to map the SVM outputs into probabilities. The mathematical framework for this model is described in detail by Platt [39]. The SMO handles multi-class (i.e. >2 groups) problems using pairwise classification. In the multi-class case the predicted probabilities are coupled using Hastie and Tibshirani's pairwise coupling method [39].

Classification accuracy was evaluated via 10 times 10-fold cross validation to ensure performance generalization. For each run of 10-fold cross validation, the data is randomly divided into 10 parts in which each class is represented in approximately the same proportions as in the full dataset. Each fold is held out in turn and the learning scheme trained on the remaining nine-tenths and the error rate is then calculated on the tenth fold. Thus the learning procedure is executed a total of 10 times on different training sets. The 10 error estimates are averaged to yield an overall error estimate. This procedure was repeated 10 times, resulting in the learning algorithm being implemented 100 times on datasets that are all nine-tenths the size of the original [31], [32]. This is a standard procedure in machine learning which reduces the variation related to data selection and allows results to be averaged to yield robust calculations of the performance of the SVM.

For the analysis of results, measures of sensitivity, specificity, accuracy and the area under the curve for the receiver operated characteristic curve (AUC ROC) are shown. Accuracy is defined as (TP+TN)/(TP+TN+FN+FP) where TP = True Positive, TN = True Negative, FP = False Positive and FN = False Negative. Sensitivity is defined as TP/(TP+FN) and Specificity is defined as TN/(FP+TN). For further details regarding SVMs and machine learning the reader is referred to the following textbook [32].

## Results

### Demographic and Cognitive Characteristics

There were no significant differences between control, MCIna and MCIa subjects in terms of age, education or MMSE (Table 1). Both MCIa and MCIna subjects performed significantly worse than controls in Verbal Fluency, Boston Naming test, Word List Average, Word Recall and Praxis. MCIa subjects performed significantly worse than MCIna subjects for Word Recall (Table 1).

**Table 1:** Demographic and Cognitive Characteristics of the Sample Groups.

Variable	Control	SD	MCIna	SD	MCIa	SD	F-value	P-value	CON>MCIa	CON>MCIna	MCIna>MCIa
	n = 40		n = 19		n = 14						
Age (years)	66	8	68	6	68	8.00	0.89	>0.05			
Gender (m/f)	16/24 (0.2)		5/14 (0.039)		7/7 (1)						
Education	13	5	12	2	13	4	0.19	>0.05			
MMSE	29.27	1.11	28.26	2.86	27.93	3.69	2.46	>0.05			
Verbal Fluency	17.60	4.02	14.26	4.75	14.36	4.61	5.24	0.01	√	√	
Boston	14.68	0.57	12.63	1.54	12.71	2.02	23.64	<0.0001	√	√	
Word List Average	7.43	1.06	6.81	1.40	5.21	2.04	13.39	<0.0001	√	√	
Word Recall	8.38	1.25	6.95	1.68	4.79	2.64	23.67	<0.0001	√	√	√
Praxis	10.63	0.70	9.89	1.33	9.36	1.91	6.74	<0.0001	√	√	
Praxis Recall	10.55	1.93	8.84	2.99	9.57	4.16	2.61	>0.05			

Values are mean ± standard deviation. Abbreviations: MCIna, non-amnestic Mild Cognitive Impairment; MCIa, amnestic Mild Cognitive Impairment; MMSE, Mini-Mental State Examination. For gender the number in brackets is the chi-square p-value. An F-value and a P-value are calculated following an anova. Post-hoc Tukey tests were performed when group differences were found with anova. Significant differences between specific groups are indicated in the far right columns (Con>MCIa, Con>MCIna, MCIna>MCIa). √ indicates the presence of significant difference.

### Differences in Multiple Indices of Diffusion between Control, MCIna and MCIa

**Figure 2:**
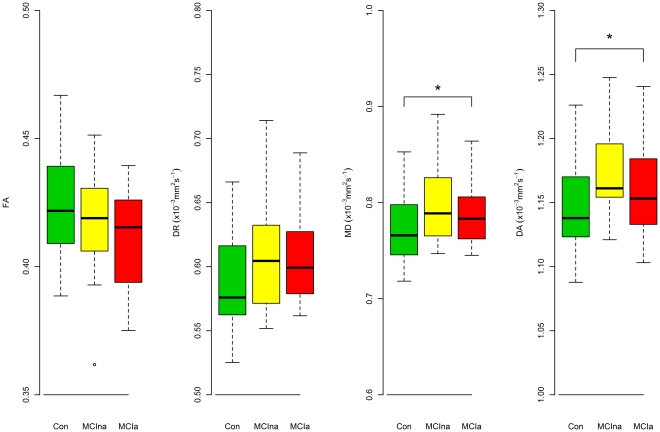
Boxplots showing the distribution of diffusion tensor MRI indices for the global WM ROI in control (CON), non-amnestic MCI (MCIna) and amnestic MCI (MCIa). The boxplots represent the interquartile ranges, which contain 50% of individual subjects' values. The whiskers are lines that extend from the box to the highest and lowest values. A line across the box indicates the median values. * p<0.05 on post-hoc Tukey test.

There were significant differences between control and MCIa groups in terms of global diffusion for MD and DA indices (Fig. 2). For FA and DR indices there were no significant differences between the groups in terms for global diffusion (Fig. 2). However, there was a trend towards higher FA values in controls relative to MCIa and MCIna in the FA index (Fig. 2). There was also trend towards lower DR values for controls relative to MCIa and MCIna subjects (Fig. 2).

### Representative Example of Data Reduction

**Figure 3:**
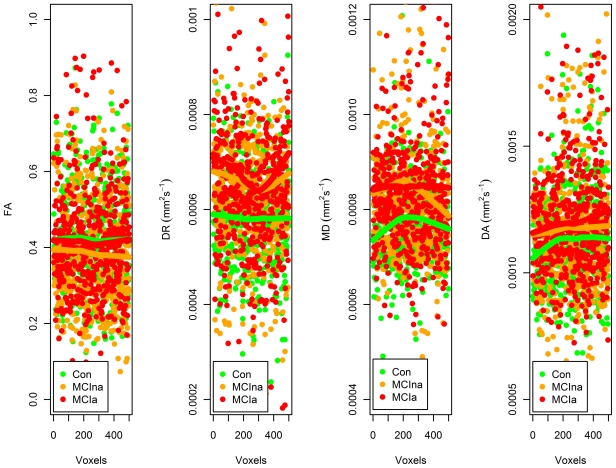
Paradigmatical reduced datasets. Following reduction of the full dataset containing diffusion values from the 130,394 voxels in the white matter skeleton, to the top 500 voxels that distinguishes between control, MCIna and MCIa subjects, this figure shows representative scatter plots from one control subject (green), one MCIna subject (orange) and one MCIa subject (red). The diffusion values for the top 500 voxels from each diffusion index are plotted. Loess regression lines (span = 2/3, polynomial degree = 1) have been fitted through each subject's dataset. For FA, the loess regression line through the data points of the control subject are seen as higher than the loess lines through the data points from MCIa or MCIna subjects. The reverse is the case for DA, DR and MD, with the loess lines through MCIa subjects indicating higher values than the lines through MCIna or control loess lines. Outliers are excluded from these graphs. For the loess line, the span which determines smoothness was set to 0.66.

A paradigmatical image of data that has been reduced using the ReliefF feature selection algorithm is shown in Figure 3. This is an example of applying ReliefF to produce the top 500 voxels for three group classification. One control, one MCIna and one MCIa subject, is chosen at random, and the FA, DA, DR and MD values within the top 500 voxels selected by ReliefF are plotted. A general profile of diffusion is seen with control subjects having the highest FA values on average, as expected. For DA, DR and MD, it can be seen that the loess line (span = 2/3, polynomial degree = 1) running through the MCIa subject shows the highest values, the MCIna subject shows intermediate values and the control subject shows the lowest values.

### SVM Classification of Control and MCI

**Figure 4:**
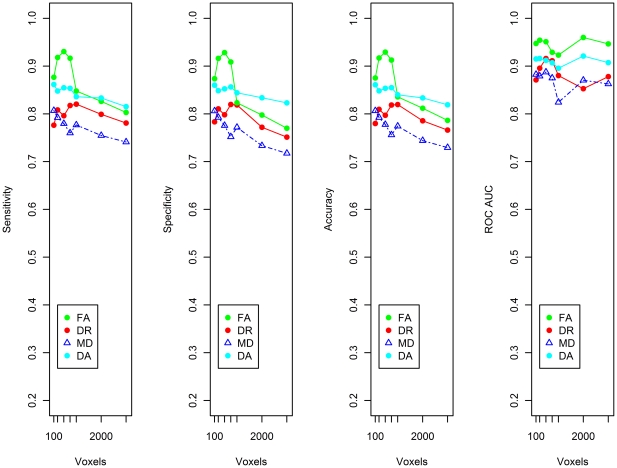
Sensitivity, specificity, accuracy and the area under the curve for a receiver operating characteristic curve (ROC AUC) for control and MCI classification. The values indicated are weighted averages for the two classes under consideration; i.e. control and MCI. Results are shown for 7 datasets – 100 voxels, 250 voxels, 500 voxels, 750 voxels, 1000 voxels, 2000 voxels and 3000 voxels. The voxels comprising these reduced datasets were selected by the ReliefF algorithm.

For the classification of control and MCI individuals, the highest sensitivity (93.0%) and specificity (92.8%) were achieved using the FA index with 500 voxel dataset (Fig. 4).

**Figure 5:**
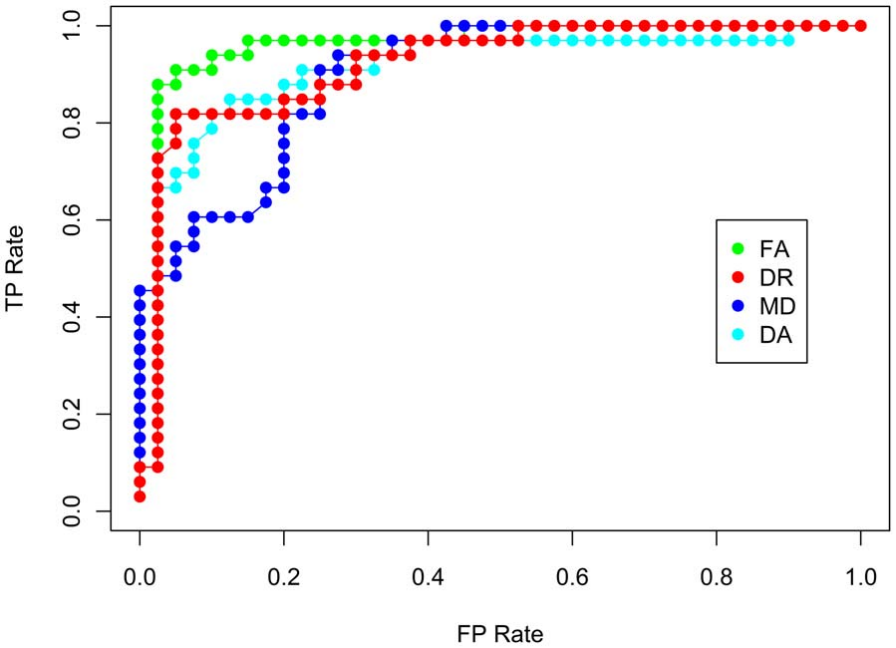
ROC curve for control and MCI classification. True positives refer to MCI volumes that are correctly classified as MCI, and false positives refer to volumes that are incorrectly labelled as MCI.

For the DA, DR and MD indices of diffusion, classification performance had a sensitivity and specificity in the range of ∼74–86% (Fig. 4). As peak performance of the SVM classifier occurs with the 500 voxel dataset, the receiver operating characteristic (ROC) curve is shown for this dataset for all 4 indices of diffusion (Fig. 5).

### SVM Classification of Control and MCIna, and MCIa

**Figure 6:**
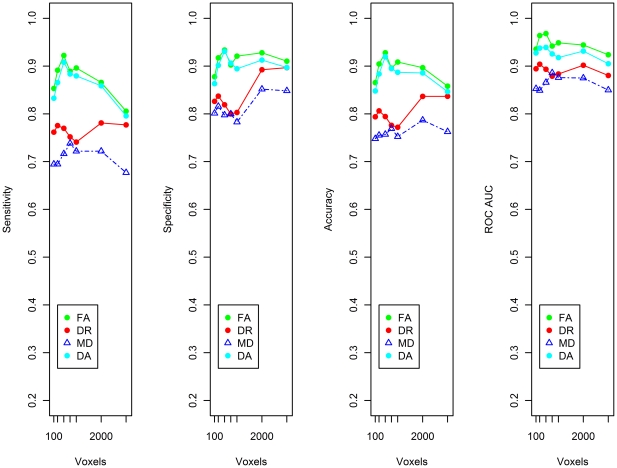
Sensitivity, specificity, accuracy and the area under the curve for a receiver operating characteristic curve (ROC AUC) for Control, MCIna and MCIa classification. The values indicated are weighted averages for the three classes under consideration; control, MCIna and MCIa. Results are shown for the 7 datasets – 100 voxels, 250 voxels, 500 voxels, 750 voxels, 1000 voxels, 2000 voxels and 3000 voxels. The voxels comprising these reduced datasets were selected by the ReliefF algorithm.

**Figure 7:**
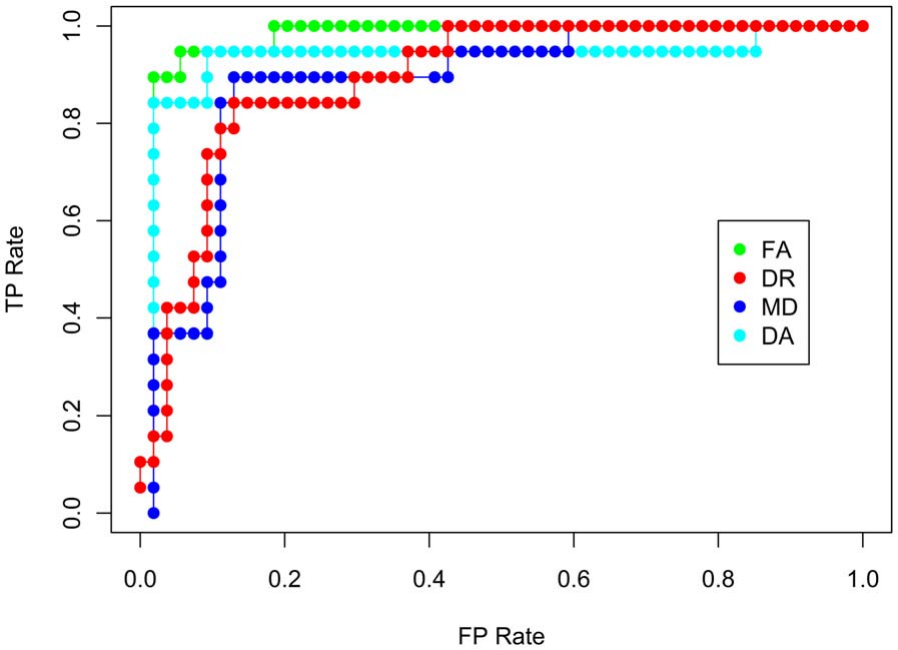
ROC curve for control, MCIna and MCIa classification. True positives refer to MCIna volumes that are correctly classified as MCIna, and false positives refer to volumes that are incorrectly labelled as MCIna.

**Figure 8:**
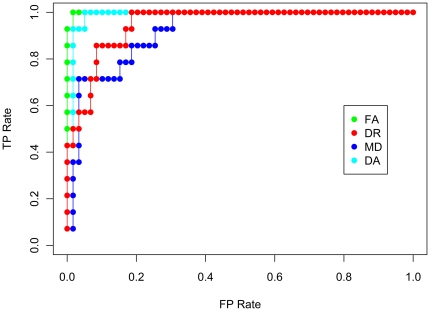
ROC curve for control, MCIna and MCIa classification. True positives refer to MCIa volumes that are correctly classified as MCIa, and false positives refer to volumes that are incorrectly labelled as MCIa.

For the control, MCIna and MCIa group classification, the best results were again obtained using the FA dataset reduced to 500 voxels. This analysis achieved maximum sensitivity of 92.2% and maximum specificity of 93.37% (Fig. 6). The ROC curve derived from the 500 voxel datasets are also shown for all four indices of diffusion. Fig. 7 depicts the ROC curve where true positive refers to a correctly identified MCIna subject and Fig. 8 depicts the ROC curve where true positive refers to a correctly identified MCIa subject.

### Regions Most influential for Classification

**Figure 9:**
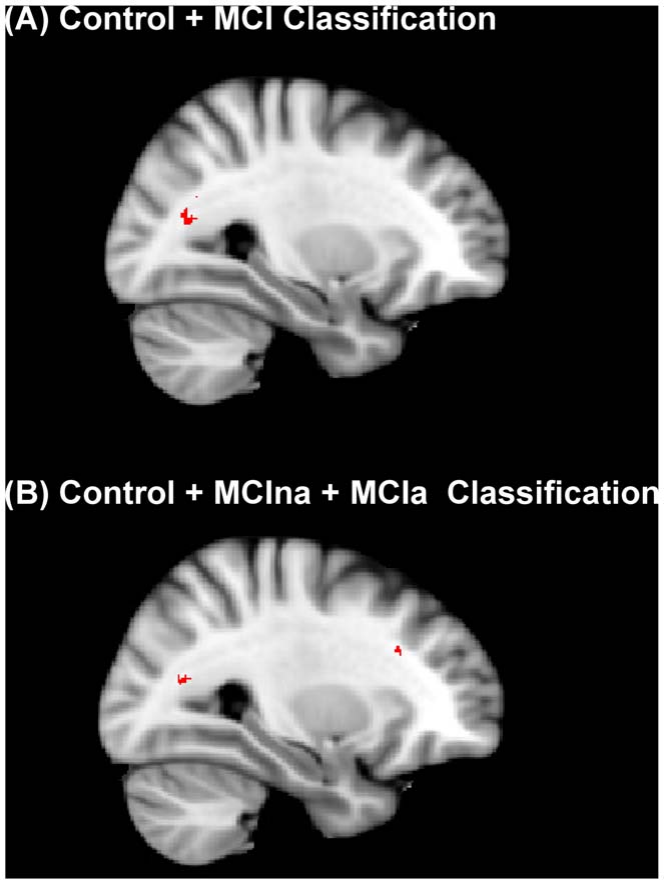
Top 500 voxels selected for classification by the Relieff algorithm. (a) Classification of control and MCI groups. The highest accuracy for this classification was achieved by the FA index. Here we show a cluster of voxels selected by the algorithm which is located in the forceps major. (b) Classification of control, MCIna and MCIa groups. For this classification of three groups, the highest accuracy was again achieved with the FA index. Here we show two significant clusters of voxels selected by Relieff. Similar to the two group classification, the forceps major was also implicated in three group classification. An additional significant cluster is located in the fronto-occipital fasciculus. Both (a) and (b) show the same sagittal slice in the right hemisphere (x = 29).

Following classification, we subsequently created images depicting the location of some of clusters of voxels selected the ReliefF algorithm. For the control versus MCI classification, a significant cluster of voxels contained within the FA dataset that produced sensitivity and specificity of 93.25 and 92.8% respectively using the top 500 voxels was visualised (Fig. 9a). In this instance, we present the largest cluster of voxels selected by ReliefF which was located in the forceps major in the right hemisphere (Fig. 9a).

For the classification of control, MCIna and MCIa subjects, the best classification performance was obtained with the FA dataset reduced to 500 voxels. Thus, two significant clusters in this dataset were visualized and shown in red (Fig. 9b). Similar to the two group classification results, a cluster was again located in the forceps major. A significant cluster was also noted in the fronto-occipital fasciculus (Fig. 9b).

## Discussion

The current results show that it is possible to classify control and MCI subjects with a high degree of accuracy using an automated procedure that combines DTI with SVMs. Our results from control versus MCI classification which achieved a sensitivity of 93.0% and specificity of 92.8% compare favourably with previous work using DTI or structural VBM data for MCI classification. The findings are extended to three group classification (control, MCIna, MCIa), with the FA index again returning the best performance with a sensitivity of 92.2% and a specificity of 93.4%. To put these results in perspective, one of the most frequently used criteria for AD classification are the NINCDS-ARDA guidelines [26] which have a sensitivity of 81% and specificity of 70% [40]. Therefore, the current automated approach adds to a growing body of evidence that MRI can be combined with machine learning algorithms to detect subtle structural damage in the early stages of Alzheimer's disease [20], [12], [13], [15], [18]. The current results are also in broad agreement with a recent SVM study which used DTI measures for the automated diagnosis of MCI subjects [36]. Wee and colleagues adopted a two stage feature selection pipeline that incorporated Pearson correlations and an SVM-RFE algorithm [41], [42]. This two stage sieving process is in contrast to the use of a single algorithm (ReliefF) for feature selection in the current study. The combined use of multiple indices of diffusion together with fiber count measures provided Wee and colleagues with an “enriched” classifier which produced an accuracy of 88% for control and MCI classification which is comparable to the accuracy achieved in the current study. Interestingly, a number of recent machine learning papers, agree with the current findings that the FA index is the optimal diffusion index for MCI and AD classification [20], [34], [36].

The current work also identifies the regions selected by the ReliefF program that are most useful for successful classification. For the classification of control and MCI groups, areas of the forceps major and the splenium were found to be particularly useful for this two group classification. Both of these regions have been shown to be compromised in MCI in previous studies [43]. This is of interest as the forceps major connects the temporal and parietal cortices and passes through the splenium [44]. This result is consistent with findings that the tempo-parietal connections may be affected in MCI via damage to the splenium. Previous studies have also found the splenium to be damaged in AD [45], [46], while in MCI, GM volume loss has consistently been localised to the medial temporal lobes and posterior cingulate [47], [48].

For the classification of three groups (control, MCIna and MCIa) ReliefF selected a significant cluster in the forceps major overlapping closely with the cluster selected for two group classification. A significant cluster in the fronto-occipital fasciculus (FOF) [49] was also identified. This also agrees with previous work that has found the FOF to be compromised in MCI and AD [50], [51]. We should stress that the ReliefF algorithm is attempting to find the most useful voxels that will aid the classification task that is defined for each particular experiment. Thus the 500 voxels that ReliefF selects for Control versus MCI classification will not be exactly the same as the 500 voxels selected for three group classification.

Joint TBSS/SVM analysis allows information to be harnessed from the entire brain, which is a significant advantage over the ROI approach that is frequently focused on the temporal lobe [52]. The current methodology obviates the need for the labour intensive selection and creation of ROIs and consequently, the approach outlined here may be suitable for use in a clinical setting. The clinical methods used by the NINCDS-ADRDA guidelines are very time consuming, while an automated approach would potentially facilitate a more efficient and objective way to streamline classification. The need for accuracy in the classification of MCI subjects is underlined by the fact that the MCIa group is at greatest risk from developing AD, while those with MCIna may progress to other forms of dementia [26]. A method which can stratify these two MCI subgroups will be of use both in the clinic and in large scale drug trials.

Also comparable to our results, a recent study has achieved accuracy rates of 90% when distinguishing control versus MCI using GM, WM and CSF volumes in conjunction with SVMs [18]. Previous PET studies have achieved 84% sensitivity at 93% specificity for the classification of control versus very mild probable AD cases [53]. PET has also been used to distinguish between AD and vascular disease with an accuracy of 80–86% accuracy [54]. Overall, our results compare favourably with accuracy rates to date, while the robustness and generality of the current method is ensured by the use of 10 times 10-fold cross-validation [32]. This method of cross validation reduces the effect of random variation when different folds are selected [31].

Some limitations of the study should be noted. In order to further validate the current findings, training and classification on multi centre data is now warranted. This is currently being pursued as part of the European DTI Study in Dementia (EDSD) initiative. For this future study the feature selection method using ReliefF will be incorporated into a nested cross-validation. While the current approach uses a feature selection framework similar to previous studies [20], this approach may be overly optimistic due to selection of features from the full dataset. The future validation of the current framework will also incorporate an assessment of a single “enriched” parameter based on a combination of all diffusion indices. The cross-sectional nature of the current data should also be noted. We do not have follow-up data and thus do not know which participants subsequently developed AD or alternatively remained stable without deteriorating further. A key aspect of machine learning in Alzheimer's disease is the distinction between progressive and stable forms of MCI. However, while such an analysis is not possible in the current cohort, a longitudinal study using the machine learning methodology outlined here is planned.

Overall, the current study demonstrates the use of DTI in conjunction with SVMs as a powerful tool for MCI classification that may be of potential use in the clinic. A fully automated procedure of this kind is an appealing alternative to cognitive batteries which are both subjective and time consuming. The pipeline outlined in the current study aims to create an SVM classifier that successfully learns the structural differences between MCI and normal healthy older people. The results are encouraging and suggest that this framework may provide a novel and efficient approach to the clinical diagnosis of mild cognitive impairment in the future.
